# Smart Surfaces: Photocatalytic Degradation of Priority Pollutants on TiO_2_-Based Coatings in Indoor and Outdoor Environments—Principles and Mechanisms

**DOI:** 10.3390/ma15020402

**Published:** 2022-01-06

**Authors:** Dimitrios Kotzias, Vassilios Binas, George Kiriakidis

**Affiliations:** 1 Former Senior Official, European Commission—Joint Research Centre, Institute for Health and Consumer Protection, 21027 Ispra, VA, Italy; 2 Institute of Electronic Structure and Laser, Foundation for Research and Technology Hellas, 100, N. Plastira Str., 71110 Heraklion, Greece; binasbill@iesl.forth.gr (V.B.); kiriakid@iesl.forth.gr (G.K.); 3 Department of Physics, University of Crete, 100, N. Plastira Str., 71110 Heraklion, Greece; 4 PCN Materials IKE, BIOPAN, Anopolis, 70008 Heraklion, Greece

**Keywords:** heterogeneous photocatalysis, indoor air, NOx, volatile organic compounds, byproducts

## Abstract

Heterogeneous photocatalysis using semiconductor oxides such as TiO_2_, provides an up-and-coming solution for the degradation of environmental pollutants compared with other technologies. TiO_2_-containing construction materials and paints activated by UV/solar light destroy the ozone precursors NO and NO_2_ up to 80% and 30%, respectively. The majority of TiO_2_ materials developed so far are primarily for outdoor use. In recent years, substantial efforts have been made to investigate further the photocatalytic activity of materials containing TiO_2_ toward priority air pollutants such as NO, NO_2_, and volatile organic compounds (VOCs) frequently accumulated at high concentration levels, particularly in indoor spaces. The intention of the investigations was to modify the titanium dioxide (TiO_2_), so that it may be activated by visible light and subsequently used as additive in building envelop materials and indoor paints. This has been achieved, to a high extent, through doping of TiO_2_ with transition metals such as V, Cr, Fe, Mn, Ni, Co, Cu, and Zn, which reduce the energy gap of TiO_2_, facilitating the generation of free electrons and holes, thus, extending the absorption spectral range of modified TiO_2_ to the area of visible light (bathochromic shift-redshift). A substantial problem using TiO_2_-containing paints and other building materials in indoor environments is the formation of byproducts, e.g., formaldehyde, through the heterogeneous photocatalytic reaction of TiO_2_ with organic matrices. This affects the air quality in confined spaces and, thus, becomes a possible risk for human health and wellbeing. This work describes the principles and mechanisms of the photocatalytic reactions at the air/catalyst interface of priority pollutants such as NO, benzene, and toluene as individual compounds or mixtures. Emphasis is placed on the reaction and recombination processes of the charge carriers, valence band positive holes (h^+^) and free electrons (e^−^), on the surface of TiO_2_, and on key factors affecting the photocatalytic processes, such as humidity. A hypothesis on the role of aromatic compounds in suppressing the recombination process (h^+^ and e^−^) is formulated and discussed. Furthermore, the results of the photocatalytic degradation of NO under visible light conditions using different admixtures of TiO_2_ and manganese doped (Mn–TiO_2_) are presented and discussed.

## 1. Introduction

Following the pioneering work of Fujishima and Honda (1972) on photocatalytic properties of TiO_2_, numerous studies were carried out to elucidate the principles, mechanisms, and mode of action of TiO_2_ [1,2,3,4]. Some of these studies aimed to evaluate the ability of TiO_2_ for environmental remediation purposes. Therefore, the development of smart coatings containing TiO_2_ as a photocatalyst have been in the foreground of research activities intending to be used in building and construction materials. The aim was the degradation of air pollutants, mainly outdoors, e.g., applied on building facades in high traffic roads, to eliminate NOx and volatile organic compounds (VOCs) [5,6,7,8,9,10,11]. However, many advantages connected with the application of TiO_2_ are limited due to its bandgap of 3.2 eV rending it efficient as a photocatalyst in the UV region (5% of the solar radiation) with wavelengths <380 nm. Further, the photocatalytic activity of TiO_2_ depends on the lifetime of the generated charged carriers—positive holes and electrons—following irradiation absorption on its surface. However, the lifetime of these species is very limited as recombination of positive holes and electrons occurs in an extremely short time (within femtoseconds) on the surface of the photocatalyst before they can react with adsorbed surface water and oxygen molecules. Thus, a primary challenge for an efficient photocatalytic process is to reduce or inhibit the recombination of charge carriers, to maintain the photocatalyst activity at a high level, increase the efficiency of TiO_2_ as a photocatalyst and expand its application. One way to reduce or inhibit recombination is to modify TiO_2_ with transition metals, which create traps for electrons and/or positive holes that block the recombination rate of charge carriers. Doping, a process that modifies the crystalline structure of TiO_2_, causes a bathochromic (red) shift due to the reduction in the energy gap of TiO_2_, thus, increasing the absorption of light and photocatalytic activity of TiO_2_ at higher wavelengths (λ > 380 nm). A number of transition metals, such as V, Cr, Fe, Mn, Ni, Co, Cu, and Zn, have been explored to reduce the energy gap facilitating the movement of electrons and, thus, extending the absorption spectral range of modified TiO_2_ towards the visible light [12,13,14,15,16]. The question remains open whether organic substances adsorbed at the TiO_2_ surface might inhibit/reduce the recombination of charge carriers (positive holes and electrons) and to what extent.

This paper describes the principles and mechanisms of the photocatalytic processes under UV and visible light illumination conditions at the air/catalyst interface. In particular, the results of the photocatalytic degradation of NO and aromatic compounds, e.g., benzene and toluene, as individual compounds or mixtures, using a commercially available TiO_2_-containing plaster board are presented and discussed. A hypothesis on the role of aromatic compounds in suppressing the recombination process (h^+^ and e^−^) at the TiO_2_ interface and the formation of free radicals is formulated and discussed. In addition, the results of the photocatalytic degradation of NO under visible light conditions using different admixtures of TiO_2_ and manganese doped (Mn–TiO_2_) are presented and discussed.

## 2. Degradation of Atmospheric Pollutants on TiO_2_-Based Photocatalytic Materials

Numerous studies have documented the degradation of NO and VOCs in TiO_2_-containing photocatalytic materials [5,6,7,8,9,10,11]. The focus of these studies was mainly on catalyst preparation and inactivation, pollutant concentration, types used as supporting materials of TiO_2_, and humidity effects. The concentration of pollutants, such as NOx, used was often very high (at ppmv levels), rarely found in polluted urban environments. TiO_2_-based construction materials (enriched with TiO_2_ at varying concentrations, typically 5 to 10%) have already been applied in buildings, pavements of squares, high traffic roads, and tunnels. The aim was to degrade NOx and possibly other pollutants emitted with the exhaust gases of automobiles using solar energy or artificial UV-light) [9,10].

**Scheme 1:**
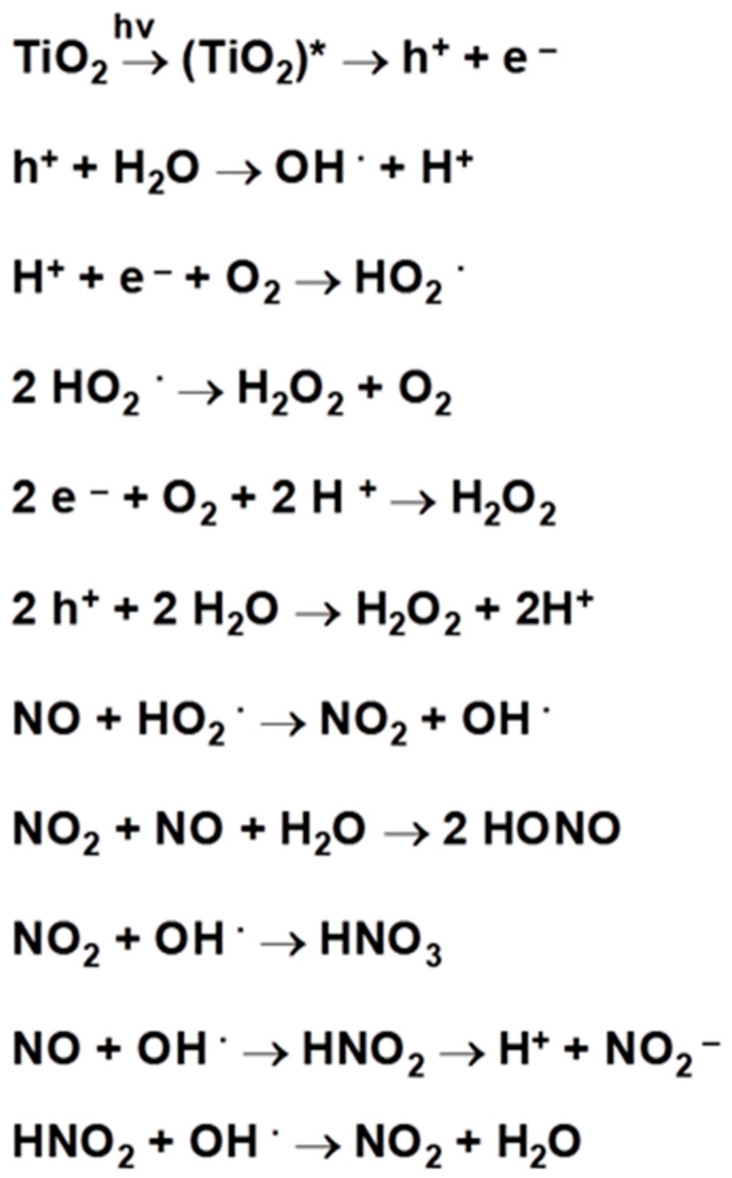
Basic Processes at the Air/Catalyst Interface by the NO degradation.

**Figure 1:**
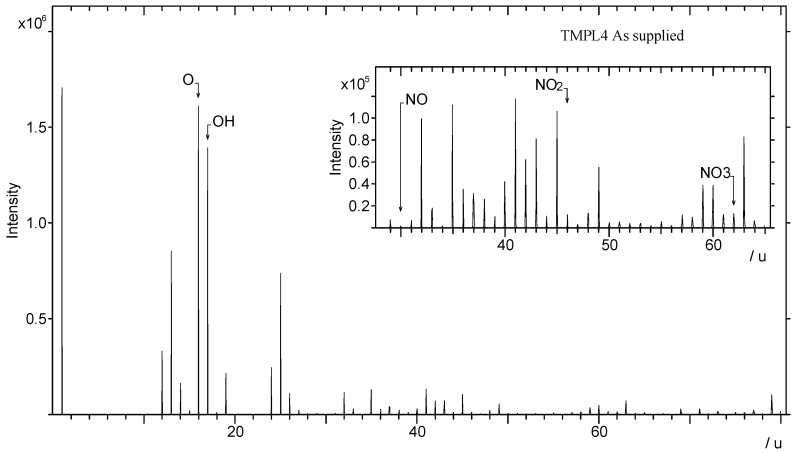
Material as supplied from the producer with TiO_2_, before the reaction of NO with UV. The sample does not show peaks assigned to NO, NO_2_, and NO_3_ after the analysis with TOF-SIMS (in mass/unit) [12].

**Figure 2:**
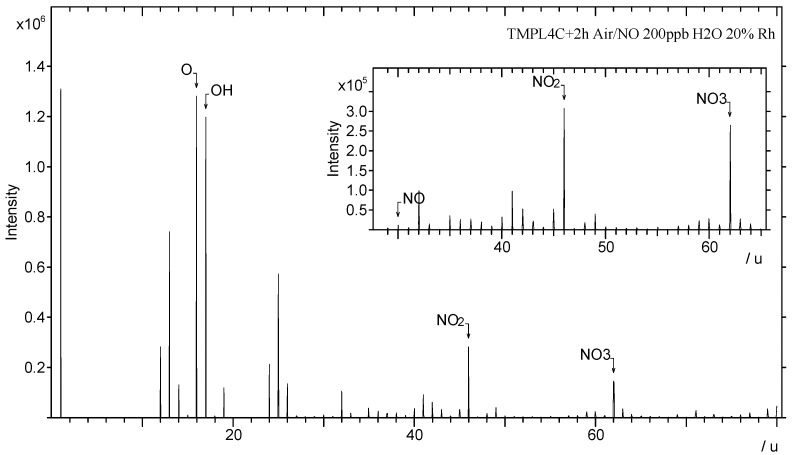
Material + TiO_2_ + UV + NO/Air/20% Rel. Humidity, after the analysis with TOF-SIMS. The sample clearly shows peaks assigned to NO_2_ (mass 46) and NO_3_ (mass 62) in mass/unit [12].

The basic chemical processes/reactions that take place at the air/catalyst interface in the presence of, e.g., H_2_O, O_2_, NO, and NO_2_ are summarized below (Scheme 1):

The photo-induced reactions that are present at the interface of air/catalyst lead to the formation of reactive radicals and oxidizing compounds that attack the NO/NO_2_ molecules. NO_2_ is formed as an intermediate product. The final products are, as expected, nitrous acid and nitric acid. The presence of nitrites and nitrates after the photocatalytic reaction has been qualitatively documented on the surface of the TiO_2_-based materials [12]. through TOF-SIMS (Time of Flight-Secondary Ion Mass Spectrometry) (Figure 1 and Figure 2).

## 3. Mixture Effects by the Degradation of NO, Benzene, and Toluene on TiO_2_-Containing Construction Material—Mechanistic Considerations

For the photocatalytic experiments presented in this study, NO, benzene, and toluene were selected as model compounds. For this purpose, a commercial TiO_2_-containing plasterboard was selected. These were tested as individual compounds or mixtures at various humidity levels to evaluate the impact and study the differences on the photocatalytic activity of TiO_2_ of mixtures of pollutants at concentrations similar to those found in the ambient air (40 ppbv for NO and 7–8 ppbv for benzene and toluene).

### 3.1. Experimental Setup

Experiments were conducted in static mode (no air exchange) in a 0.45 m^3^ glass chamber, equipped with fans to ensure internal air mixing. Temperature, relative humidity, as well as the quality of air in the chamber, were continuously controlled. The operating temperature was fixed at 23 °C, while the relative humidity was fixed to 20% or 60%. Temperature and relative humidity values throughout the whole experiment were monitored by means of Escort RH iLog data loggers (Escort, New Lynn, Auckland, New Zealand). The initial levels of NO (40 ppbv), benzene, and toluene (7–8 ppbv) in the chamber were achieved by direct injection of a known amount of the individual chemicals taken from certified gas cylinders with the concentrated compounds.

The photocatalytic material was irradiated for six hours with a UV exposure unit consisting of one OSRAM UltraVitalux 300 W lamp. Light intensities over the material surface in the visible and UV-A spectral regions were measured with a Delta Ohm HD9021 radiometer of 31.8 and 4.1 W/m^2^, respectively. Light intensities in both the UV-B and UV-C spectral regions were negligible.

### 3.2. Analysis of NO, Benzene, and Toluene

The concentration of the target pollutants inside the chamber was monitored on a regular basis. NO and NOx were analyzed online by means of a chemiluminescent NO/NOX gas analyzer (Thermo Environmental Instruments), while VOC concentration was determined offline. For this purpose, 2 L of the chamber air were drawn through Car-bopack-B thermal desorption tubes (Supelco) at a flow rate of 100 mL/min. The samples were subsequently analyzed by thermal desorption (Perkin Elmer TD 650) coupled to a gas chromatograph (Agilent Technologies 7890A), equipped with a DB-5-MS column (J & W Scientific, L 30 m, D 0.25 mm, 1 µm film), with a mass spectrometer as detector (Agilent Technologies 5975C). Helium was used as carrier gas at a flow rate of 1.5 mL/min. The oven temperature was programmed from 40 °C to 280 °C at 15 °C/min.

Thermal desorption of benzene and toluene was completed by heating the Carbopack tube at 280 °C for 10 min. Desorbed benzene and toluene were transferred with a helium gas stream of 50 mL/min and enriched on a cold trap packed with 100 mg Tenax TA set at −30 °C. Subsequent desorption from the cold trap was performed by rapidly heating up to 280 °C. Benzene and toluene were directly transferred into the gas chromatographic column.

### 3.3. Photocatalytic Experiments

A commercial gypsum-based thin layer plaster, containing TiO_2_ as a photocatalyst, was tested for the degradation of NO, benzene, and toluene at the ppbv level. Prior to the experiments, the sample was introduced into the environmental chamber and conditioned by passing zero air at an air exchange rate of 1 h^−1^ for 18 h. In order to assess the influence of other depletion mechanisms that could compete with photocatalysis, the possible adsorption of the pollutants onto the chamber walls and the photolytic degradation were estimated in each experiment as well as on the photocatalytic material in the dark. Irradiation time during the photocatalytic experiments was fixed to six hours.

## 4. Photocatalysis of NO under Indoor-Like Illumination Conditions

Details on the experimental setup are given elsewhere [13]. In short, thirty grams of the photocatalyst powder (0.1% and 1% doped Mn–TiO_2_) was spread homogeneously in a 0.1 m radius Petri dish and placed in a 0.45 m^3^ environmental test chamber in which a controlled atmosphere containing approximately 200 ppbv NO was created. Furthermore, samples of calcareous filler containing 0%, 5%, or 10% of the 0.1% doped Mn–TiO_2_ photocatalyst were deposited on 0.25 m × 0.25 m glass panels. The photocatalytic tests were carried out by irradiating the photocatalyst for 6 h, both in powder form and embedded in the calcareous matrix. During this period, the concentration of both NO and NOx in the chamber was analyzed on a periodic basis.

## 5. Results and Discussion

### 5.1. Mixture Effects—Mechanistic Considerations

**Figure 3:**
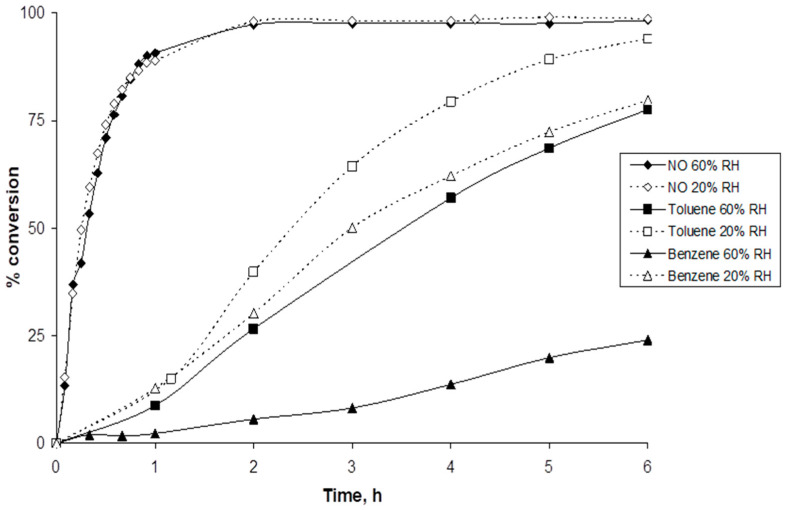
Influence of humidity on the photocatalytic degradation of NO, benzene, and toluene.

**Figure 4:**
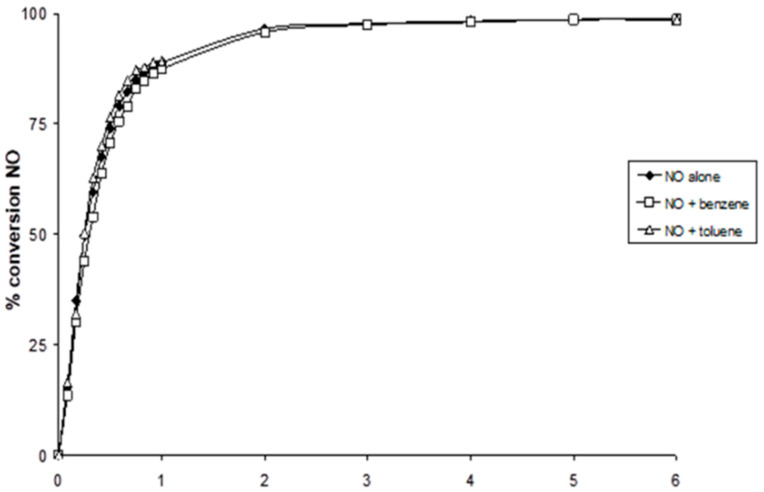
Photocatalytic degradation of NO alone and in the presence of benzene and toluene (RH: 20%). A similar result was obtained at RH 60%.

Following three hours of irradiation with UV-light under the experimental conditions in this study, nearly 100% of NO degraded at both humidity levels (20 and 60%), while the photocatalytic degradation of benzene and toluene in the same time period remained (at RH 60%) below 50%. At 20% RH, the degradation of benzene and toluene, after three hours, was 50% and 63%, respectively (Figure 3). It was found that neither benzene nor toluene had any influence on the photocatalytic degradation of nitrogen oxide (NO) at any of the humidity levels applied in the experiments (Figure 4). It should be mentioned that the degradation of NO proceeds not only heterogeneously on the TiO_2_ surface, but also in the gas phase via the oxidation with oxygen (NO + 1/2 O_2_ -> NO_2_ + O) to form NO_2_. The elimination of NO_2_ from the gas phase through adsorption on the TiO_2_-containing materials is a speed-determining step for the NO oxidation to NO_2_.

**Figure 5:**
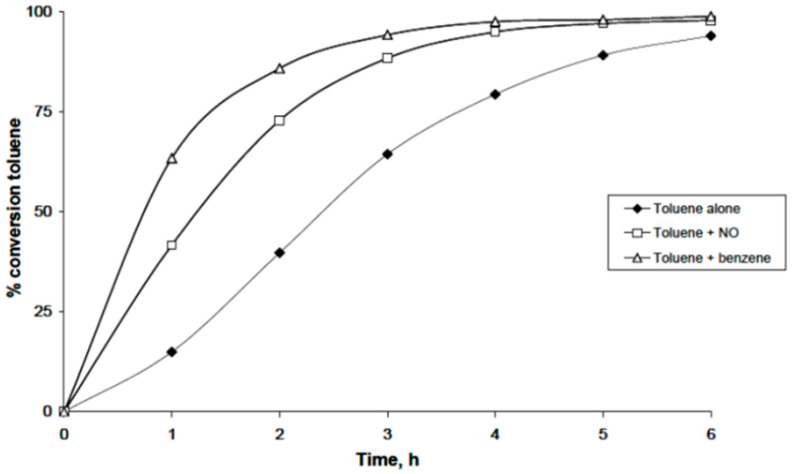
Photocatalytic degradation of toluene alone and in the presence of NO and benzene at RH 20%.

**Figure 6:**
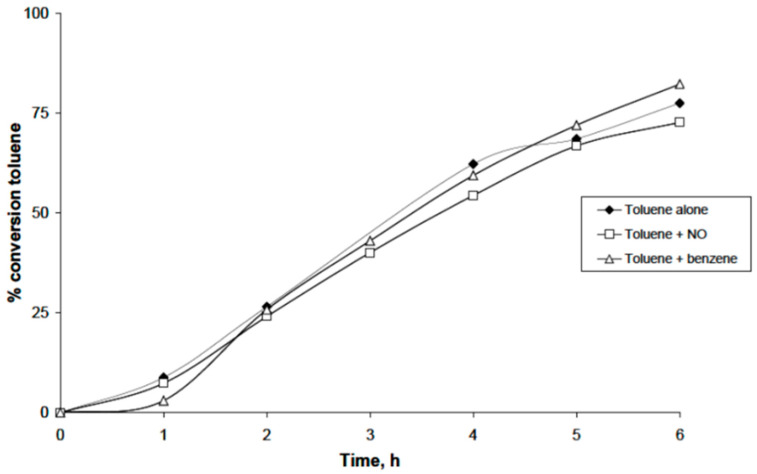
Photocatalytic degradation of toluene alone and in the presence of NO and benzene at RH 60%.

The photocatalytic degradation of toluene (in mixtures with NO and benzene) after three hours of irradiation, was strongly influenced by the presence of benzene and nitrogen oxide at 20% relative humidity, while at humidity levels of 60% the degradation of all three model compounds remained below 50%. The impact of nitrogen oxide on the degradation of benzene and toluene at the 60% humidity level was found to be negligible (Figure 5 and Figure 6).

**Figure 7:**
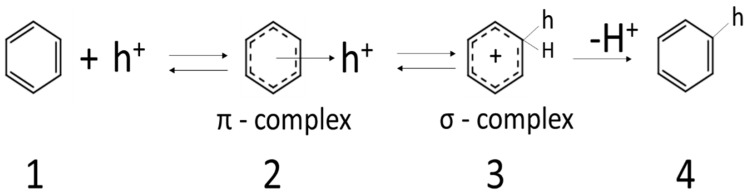
The electrophilic mingling of a positive hole (h^+^) with the benzene ring. Step 2 represents the mesomeric/resonance forms in the benzene ring.

The fact that benzene promotes the photocatalytic degradation of toluene (at RH 20%) in the photochemical system without the presence of NO is interesting. It cannot be explained through the generation of OH radicals according to the oxidation of water following its interaction with positive holes. Both compounds benzene and toluene react with the OH-radicals, while the rate constant of the reaction OH-toluene is greater (5.78 × 10^−12^ cm^3^/mol s) than the rate constant of benzene (12.9 × 10^−13^ cm^3^/mol s). Thus, when both compounds (benzene and toluene) are present in the photo-chemical system, the generated OH-radicals are available and react (even with different rate constants) with both compounds; the OH radicals are not entirely available for one compound only. This means that in the case of the irradiation of toluene as a single compound, the photocatalytic degradation would be faster than in the case of the irradiation of a mixture with benzene, because OH-radicals react with or are consumed by one compound, namely toluene, only. For example, after two hours of irradiation the degradation of toluene as a single compound was 40%, while in the same time the degradation of toluene in the presence of benzene reached values of up to 85% (Figure 5). Preliminary evidence indicated that in the benzene/toluene photochemical system, the benzene with its delocalized p-orbital setup in the molecule and toluene, more reactive than benzene toward electrophiles, under the impact of UV-light are acting as electron suppliers to the positive holes (electrophiles). Comparing the ionization potential of H_2_O (12.65 eV), benzene (9.24 eV), and toluene (8.81 eV), it can be expected that positive holes are trapped by electrons originating from the aromatic molecules rather than from water molecules (at 20% RH), and the oxidation of the aromatic compounds initially proceeds via the valence band holes. The overall process is similar to the classical electrophilic substitution of aromatic compounds (Figure 7). The reactions 1, 2, and 3 that take place on the TiO_2_ surface in the presence of benzene and toluene contribute to the overall generation of radicals and oxidizing agents (H_2_O_2_). Assuming the predominant degradation of aromatics proceeds via their reaction with OH radicals, preliminary calculations indicate that a substantial amount of OH radicals formed in the benzene/toluene photochemical system at low humidity level (20% RH) originates from the direct interaction of the aromatic compounds with the positive holes. This leads to a higher degradation of toluene, compared to the degradation of the same compound in the toluene/NO system or by the irradiation of toluene as an individual compound. Targeted experiments with various mixtures of aromatic compounds could provide further clarification on this issue.

**Scheme 2:**
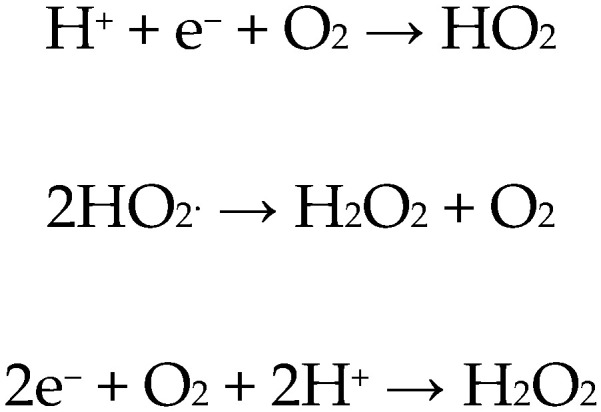
Reactions take place at the air/catalyst interface following the release of a H+ from the aromatic ring.

Based on the experimental results obtained, our analysis of the surface processes (Scheme 2) indicates that reactive species such as OH and HO_2_ radicals as well as other oxidizing compounds (H_2_O_2_) cannot be photochemically generated via hole-trapping of water only. It is questionable whether positive holes have enough potential to oxidize adsorbed water molecules rather than aromatic compounds, which have a lower ionization potential than water.

### 5.2. Heterogeneous Photocatalysis of NO under Indoor-Like Illumination Conditions 

TiO_2_ modified/doped with 0.1% (w/w) manganese is capable of degrading NO up to 95% under indoor illumination conditions, while the 1% Mn modified/doped photocatalyst was active with solar radiation only. The results indicate that not only the presence of manganese but also its concentration in the crystalline structure of TiO_2_ plays a significant role for the photocatalytic reaction in the visible region of the spectrum. Besides, results from addition of 5% and 10% (w/w) of the 0.1% Mn–TiO_2_ photocatalyst to calcareous filler commonly used in the formulation of building products indicate its efficiency, in this form too, to degrade NO at concentration levels typically found in indoor and outdoor environments [13].

### 5.3. Byproducts: Emission of Low Molecular Weight Carbonyls by the Irradiation of TiO_2_-Enriched Paints

**Figure 8:**
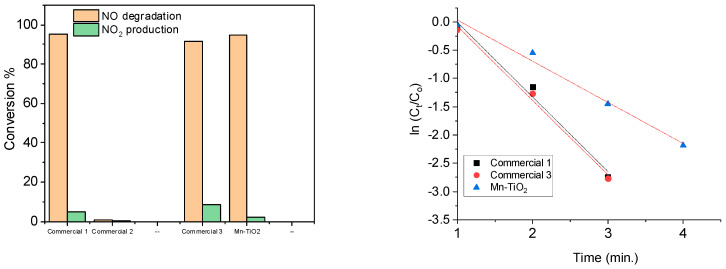
Degradation of NO under visible light illumination utilizing a number of commercial products.

Many companies have developed construction and building envelop materials, particularly paints, enriched with pure TiO_2_ or doped TiO_2_ to degrade priority air pollutants in outdoor and indoor environments. The ultimate goal has been to create materials (paints) that degrade priority pollutants that do not represent a risk for human health, mainly when used in confined spaces. However, there have been some strong indications of emissions, from TiO_2_-based building paints after irradiation, of low molecular weight carbonyls, e.g., formaldehyde (HCHO) and acetaldehyde (CH_3_CHO). This fact makes the use of TiO_2_-paints in indoor environments problematic. In addition, a recent study on the degradation of NO under visible light illumination (LED: 460 nm, 10 W) utilizing a number of commercial products has shown that while the degradation of NO was of the order of >80% for the most effective photocatalysts, the amount of NO_2_ production varied significantly from product to product. In particular, while the Mn–TiO_2_ material exhibited a conversion efficiency of NO to NO_2_ of the order of about 2% some of the most well-known commercial counterparts exhibited two-fold and four-fold higher values, i.e., 4% and 8% correspondingly (Figure 8).

Based on the above, findings on wet paints indicate as a source for carbonyl compounds the reaction of TiO_2_ with the various components/additives [17], while the degradation of an inorganic pollutant may hide the formation of an unacceptable level of harmful byproduct formation. Thus, the subject of byproduct formation, often overlooked, should become an important parameter in the overall assessment, particularly of commercial products targeted for indoor applications Therefore, the development of photocatalytic coatings aimed to be applied to indoors needs, should always be optimized to minimize the emission of harmful substances.

## 6. Conclusions

The degradation of NOx gases and volatile organic compounds (VOCs) at concentrations typical for urban/indoor environments using heterogeneous photocatalysis (based on TiO_2_ semiconductors) has been presented. Humidity was found to affect the photo-degradation of NOx and VOCs. Increasing humidity at high NO concentration levels reduced the catalyst activity due to the competition of water molecules with molecules of other compounds for the adsorption sites.

A hypothesis on the role of aromatic compounds as possible inhibitors of the recombination process (h^+^ and e^−^) was formulated and discussed.

TiO_2_-containing photocatalytic paints, especially for indoor environments, need to be optimized (concerning individual organic paint components) to reduce or eliminate toxic compounds, which might be produced through the photocatalytic process.

By selecting both the appropriate dopant type (in our experiments Mn) and its concentration in the crystal lattice of TiO_2_, heterogeneous photocatalysis under indoor illumination conditions could be a promising technique for the degradation of pollutants in indoor environments.

## Data Availability

Not applicable.
